# Divergent roles of prostacyclin and PGE_2_ in human tendinopathy

**DOI:** 10.1186/s13075-019-1855-5

**Published:** 2019-03-13

**Authors:** Filip Bergqvist, Andrew J. Carr, Kim Wheway, Bridget Watkins, Udo Oppermann, Per-Johan Jakobsson, Stephanie G. Dakin

**Affiliations:** 1 Rheumatology Unit, Department of Medicine, Solna, Karolinska Institutet, Karolinska University Hospital, SE-17176 Stockholm, Sweden; 2 Nuffield Department of Orthopaedics, Rheumatology and Musculoskeletal Sciences, Botnar Research Centre, University of Oxford, Nuffield Orthopaedic Centre, Headington, OX3 7LD UK; 3 Structural Genomics Consortium, University of Oxford, Old Road Campus, Headington, OX3 7DQ UK

**Keywords:** Tendinopathy, Inflammation, Pain, Prostacyclin, PGE_2_

## Abstract

**Background:**

Tendon disease is a significant global healthcare burden whereby patients experience pain and disability; however, the mechanisms that underlie inflammation and pain are poorly understood. Herein, we investigated the role of prostaglandins as important mediators of inflammation and pain in tissues and cells derived from patients with tendinopathy.

**Methods:**

We studied supraspinatus and Achilles tendon biopsies from symptomatic patients with tendinopathy or rupture. Tendon-derived stromal cells (CD45^neg^CD34^neg^) isolated from tendons were cultured and treated with interleukin-1β (IL-1β) to investigate prostaglandin production.

**Results:**

Diseased tendon tissues showed increased expression of prostacyclin receptor (IP) and enzymes catalyzing the biosynthesis of prostaglandins, including cyclooxygenase-1 (COX-1), COX-2, prostacyclin synthase (PGIS), and microsomal prostaglandin E synthase-1 (mPGES-1). PGIS co-localized with cells expressing Podoplanin, a marker of stromal fibroblast activation, and the nociceptive neuromodulator NMDAR-1. Treatment with IL-1β induced release of the prostacyclin metabolite 6-keto PGF_1α_ in tendon cells isolated from diseased supraspinatus and Achilles tendons but not in cells from healthy comparator tendons. The same treatment induced profound prostaglandin E_2_ (PGE_2_) release in tendon cells derived from patients with supraspinatus tendon tears. Incubation of IL-1β treated diseased tendon cells with selective mPGES-1 inhibitor Compound III, reduced PGE_2_, and simultaneously increased 6-keto PGF_1α_ production. Conversely, COX blockade with naproxen or NS-398 inhibited both PGE_2_ and 6-keto PGF_1α_ production. Tendon biopsies from patients in whom symptoms had resolved showed increased *PTGIS* compared to biopsies from patients with persistent tendinopathy.

**Conclusions:**

Our results suggest that PGE_2_ sustains inflammation and pain while prostacyclin may have a protective role in human tendon disease.

**Electronic supplementary material:**

The online version of this article (10.1186/s13075-019-1855-5) contains supplementary material, which is available to authorized users.

## Introduction

Inflammatory and fibrotic diseases of the joint are a significant cause of pain and impaired physical function [1]. Inflammatory processes are increasingly recognized to contribute to the onset and progression of tendinopathy [2, 3], although the precise mechanisms driving inflammation and pain associated with disease remain to be fully elucidated. Current therapeutic regimens include non-steroidal anti-inflammatory drugs (NSAIDs), glucocorticoids, or use of biological therapies including platelet-rich plasma in association with prolonged physical rehabilitation. However, long-term use of glucocorticoids or NSAIDs is associated with adverse effects including tendon rupture [4–6] and impaired healing [7, 8]. Surgical repair is frequently required in patients with tendon tears; however, postoperative failure rates occur in up to 40% of patients [9]. Improved understanding of the biological mechanisms driving inflammation and pain are therefore required to inform development of effective new treatments targeting cells driving tendon disease.

Prostaglandins are key lipid mediators regulating physiological functions and inflammatory responses in health and disease [10]. They are synthesized from arachidonic acid via cyclooxygenases (COX-1 and COX-2) to generate unstable prostaglandin H_2_, which is then further processed by terminal synthases into the major prostaglandins (prostacyclin, PGE_2_, PGD_2_, and PGF_2α_) or thromboxane A_2_ (TXA_2_) [11]. Prostacyclin synthase (PGIS) is highly expressed in vascular endothelial and smooth muscle cells. Prostacyclin is a potent regulator of vascular tone by dilating vessels and inhibiting platelet aggregation [12] but it is also a mediator of inflammatory and pain responses [13]. Microsomal prostaglandin E synthase-1 (mPGES-1) is the essential terminal enzyme for biosynthesis of PGE_2_. In line with COX-2, mPGES-1 is induced in cells including fibroblasts and macrophages upon exposure to pro-inflammatory stimuli. PGE_2_ is implicated in all processes leading to the cardinal signs of inflammation, including heat, redness, swelling, and pain [12], and is an immune modulator via actions on lymphoid and myeloid cells [14]. NSAIDs, including NS-398 and naproxen, are frequently used to treat pain and inflammation due to their COX-specific reduction in prostaglandin biosynthesis. However, long-term use of NSAIDs are associated with severe systemic side effects, such as gastrointestinal bleeding, decreased renal function, and increased risk of cardiovascular events and stroke [15]. Using NSAIDs to relieve pain in tendinopathy is controversial as these treatments are associated with impaired tendon healing [7, 8] and adverse effects on tendon mechanical properties [16]. Therefore, inhibition of the downstream enzyme in the PGE_2_ biosynthesis is a potential strategy to selectively target inflammation without the deleterious side effects associated with COX inhibition [17].

Inflammation is a highly coordinated process, involving interplay between cells of the innate immune system and resident tissue stromal cells including fibroblasts. Previous studies have identified the complex activation states of myeloid cells in tissue biopsies from patients with tendinopathy [18]. Resident tendon stromal fibroblasts are also implicated in sustaining chronic inflammation, as diseased tendon tissues and cells highly express markers of stromal fibroblast activation including Podoplanin (PDPN), vascular adhesion molecule 1 (VCAM-1 or CD106), and endosialin (CD248) [19]. It is well established in the literature that a growing list of cell surface molecules including PDPN collectively make up a panel of fibroblast activation markers that are highly expressed under inflammatory conditions [20, 21]. These markers represent important phenotypic alterations that have been implicated in effecting the switch from resolving inflammation to persistent inflammation [22]. Improved understanding of how resident stromal fibroblasts (the major cell type in tendons) regulate inflammation and pain processes is essential to advance therapeutic target discovery to treat common joint diseases.

The present study focused on identifying prostaglandins mediating inflammation and pain in tissue samples collected from patients with tendinopathy. We investigated expression of enzymes implicated in the production of prostaglandins including prostacyclin and PGE_2_ in tendon tissues collected from patients with tendinopathy compared to those from healthy volunteers. Using cultures of tendon stromal fibroblasts derived from healthy and diseased human tendons, we next investigated differences in the prostaglandin profiles of these cells in the presence or absence of interleukin-1β (IL-1β). We subsequently determined if COX and mPGES-1 inhibitors moderated prostaglandin profiles. Finally, we determined *PGIS* mRNA expression in tendon tissue biopsies collected from pain-free patients and those with persistent pain after surgical treatment. The findings from this study advance understanding of the divergent roles of PGE_2_ and prostacyclin in tendon disease, identifying these mediators as druggable therapeutic targets.

## Methods

### Shoulder tendon cohorts

All patients were recruited from orthopedic referral clinics where the structural integrity of the rotator cuff was determined ultrasonographically and the presence or absence of a supraspinatus tendon tear identified. Patients with shoulder tendon disease completed the Oxford Shoulder Score (OSS), a validated and widely used clinical outcome measure scoring from 0 (severe pathology) to 48 (normal function). Presenting patients with shoulder tendon tears had failed non-operative treatment and experienced pain for a minimum of 3 months. Supraspinatus tendon tear samples were collected at the time of surgical debridement of the edges of the torn tendons from four male and four female patients aged between 55 and 68 years. All patients were symptomatic and had small to medium tendon tears (≤ 1 cm to ≤ 3 cm in anterior-posterior length). Exclusion criteria included previous shoulder surgery, other shoulder pathology, systemic inflammatory disease, and rheumatoid arthritis.

Tissue biopsies of early-stage pre-treatment tendinopathic (not torn) supraspinatus were taken from patients undergoing arthroscopic subacromial decompression (ASAD) surgery (painful pre-treatment group, *n* = 5). These patients exhibited tendon pain and loss of function (demonstrated in their OSS) in the absence of a structural tendon tear. Biopsies were also taken from patients between 2 and 4 years after ASAD surgery, in whom pain had resolved completely (pain-free post-treatment, *n* = 6) or pain persisted (painful post-treatment, *n* = 5). Pain-free post-treatment patients had significant pain before their surgery was performed as evidenced by a median OSS of 24 (range, 20 to 40) before surgery. All tendinopathic patients were of ages between 38 and 65 years and had not received cortisone treatment for 3 months prior to inclusion in the study. Tendon biopsies were collected under ultrasound guidance and local anesthetic, using a previously validated biopsy technique [23].

### Achilles tendon cohort

All patients were recruited from orthopedic referral clinics where the structural integrity of the Achilles tendon was determined ultrasonographically. Tendon tissues were collected from patients with Achilles tendinopathy (*n* = 3) or acute traumatic rupture (*n* = 3) aged between 44 and 64 years. Tissue biopsies were collected from four male and two female patients with Achilles tendinopathy using a 14G trucut biopsy needle inserted into the mid portion of the Achilles under ultrasound guidance. Achilles tendon ruptures were collected from patients between 36 and 48 h after tendon rupture during surgical debridement of the tendon edges.

### Healthy tendon cohort

Comparator healthy hamstring tendons were collected from six male and two female patients undergoing surgical reconstruction of their anterior cruciate ligament. All patients were aged between 22 and 48 years. Exclusion criteria for hamstring donors included the presence of hamstring tendinopathy or previous hamstring surgery. Patients with systemic inflammatory disease and inflammatory arthritis were also excluded.

### Immunostaining for enzymes implicated in prostaglandin production in healthy and diseased tendons

Tendon samples were immersed in 10% buffered formalin, processed using a Leica ASP300S tissue processor, and embedded in paraffin wax. Tissues were sectioned to 4 μm onto adhesive glass slides. For antigen retrieval, slides were baked at 60 °C for 60 min and tissue sections were taken through deparaffinization and target retrieval steps (high pH heat-mediated antigen retrieval) using an automated PT Link (Dako, UK). For single-staining immunohistochemistry, antibody staining was performed using the EnVision FLEX visualization system with an Autostainer Link 48 (Dako). Antibody binding was visualized using FLEX 3,3′-diaminobenzidine (DAB) substrate working solution and hematoxylin counterstain (Dako) using the recommended manufacturer protocols. After staining, slides were taken through graded alcohol and xylene and mounted in Pertex mounting medium (Histolab, UK). For multiple antibody immunofluorescence staining and image acquisition, protocols were adapted from [18] using primary antibodies listed in Table 1. Isotype control antibodies were a cocktail of mouse immunoglobulin G (IgG_1_), IgG_2a_, IgG_2b_, IgG_3_, and IgM (Dako) and rabbit immunoglobulin fraction of serum from non-immunized rabbits, solid-phase absorbed (Dako). Immunofluorescence images were acquired on a Zeiss LSM 710 confocal microscope using a × 40 oil immersion objective (NA = 1.3) using previously described protocols [18].

**Table 1:** List of primary antibodies used. Antibody for mPGES1, in-house [54]

Antigen	Antibody ID	Isotype	Species	Dilution
IP receptor	LS Bio, cat# LS-A4395	IgG	rabbit	1:100
PGIS	LS Bio, cat# LS-C167246	IgG	rabbit	1:100
CD68	Dako, cat# M0814	IgG_1_	mouse	1:100
CD31	Abcam, cat# ab187377	IgG_1_	mouse	1:150
CD34	Proteintech, cat# 60180	IgG_2b_	mouse	1:100
COX-2	Abcam, cat# ab10940	IgG_1_	mouse	1:100
mPGES-1	In house (Westman et al., 2004)	IgG	rabbit	1:5000
COX-1	LS Bio, cat# LS-C14603	IgG_2b_	mouse	1:100
PDPN	Abcam, cat# ab10288	IgG_1_	mouse	1:100
NMDAR-1	BD Biosciences, cat# BD556308	IgG_2a_	mouse	1:250
TLR4	Abcam, cat# ab22048	IgG_2b_	mouse	1:200
IL-1R	Abcam, cat# ab190078	IgG	rabbit	1:200

### Isolation of tendon-derived stromal cells from healthy and diseased tendons

Tendon-derived stromal cells were isolated using previously published protocols [18]. Our previous characterization of human tendon-derived cells using fluorescence-activated cell sorting (FACS) demonstrated cells used for in vitro experiments were CD45^neg^ and CD34^neg^ [19]. This was confirmed in this study using antibodies towards CD45 (BV605 mouse IgG1 anti-human, BioLegend, cat# 304041) and CD34 (PerCP/Cy5.5 mouse IgG1 anti-human, BioLegend, cat# 343521), both at 1:50 dilution, according to previously published protocol [19].

### Treatment of tendon-derived stromal cells with IL-1β to induce prostaglandin release

As IL-1β induces nuclear factor kappa beta (NF-κB) target genes known to be highly expressed in early-stage tendinopathy [18], we performed profiling of prostaglandins in cells derived from healthy and diseased human tendons in the presence and absence of IL-1β. Tendon-derived stromal cells from healthy hamstring (*n* = 8), diseased supraspinatus tendons (*n* = 8), and diseased Achilles tendons (*n* = 6) were seeded at a density of 60,000 cells per well in a six-well plate. Tendon cells were allowed to reach 80% confluence prior to treatment with IL-1β (Merck, 10 ng/mL) in DMEM F12 medium (Lonza, UK) containing 1% heat-inactivated human serum (Sigma) and 1% penicillin-streptomycin. Non-treated cells (vehicle only, containing 0.1% endotoxin-free BSA, Sigma) served as controls for each experiment. After treatment, cells were then incubated at 37 **°**C and 5% CO_2_ until harvest of the supernatant for prostaglandin profiling after 24 h. Samples were stored at − 80 °C prior to analysis.

### Treatment of tendon-derived stromal cells with inhibitors for COX or mPGES-1

The cyclooxygenase-1/2 (COX-1/2) inhibitor naproxen and the selective COX-2 inhibitor NS-398 were purchased from Sigma Aldrich (UK). The mPGES-1 inhibitor Compound III (CIII) [24] was synthesized by NovaSAID AB (Solna, Sweden). The inhibitors were reconstituted in DMSO. Tendon-derived stromal cells from healthy hamstring (*n* = 4) and diseased supraspinatus tendons (*n* = 4) were seeded and treated with IL-1β according to above. At the time of IL-1β treatment, cells were co-treated with 10 μM of naproxen, NS-398, or CIII. Medium containing IL-1β and 0.1% DMSO (vehicle) served as control for each experiment. Cells were then incubated at 37 **°**C and 5% CO_2_ until harvest of the supernatant for prostaglandin profiling after 24 h. Samples were stored at − 80 °C.

### Prostaglandin profiling using LC-MS/MS

Prostaglandins in cell supernatants were extracted and analyzed with liquid chromatography tandem mass spectrometry (LC-MS/MS) for absolute quantification. Supernatants (450 μL) were thawed on ice and spiked with 50 μL deuterated internal standards of 6-keto PGF_1α_-d4, PGF_2α_-d4, PGE_2_-d4, PGD_2_-d4, TXB_2_-d4, and 15-deoxy-Δ12,14PGJ_2_-d4 (Cayman Chemical Company) in 100% methanol. Samples were made acidic with 50 μL 1% formic acid (FA) in water and incubated on ice for 30 min. Solid-phase extraction (SPE) was performed by loading samples on Oasis HLB 1 cc 30 mg cartridges (Waters Corporation, MA, USA) that had been preconditioned with 100% methanol and 0.05% FA in water. The SPE columns were washed once with 10% methanol in water followed by elution in 100% methanol. The samples were evaporated to dryness under vacuum and stored at − 20 °C until reconstituted in 50 μL of 20% acetonitrile prior to analysis with liquid chromatography tandem mass spectrometry (LC-MS/MS). Analytes were quantified in negative mode with multiple reaction monitoring method, using a triple quadrupole mass spectrometer (Acquity TQ detector, Waters) equipped with an Acquity H-class UPLC (Waters). Separation was performed on a 50 × 2.1 mm Acquity UPLC BEH C18 column 1.7 μm (Waters) with a 12-min stepwise linear gradient (20–95%) at a flowrate of 0.6 mL/min with 0.05% FA in acetonitrile as mobile phase B and 0.05% FA in water as mobile phase A. Data were analyzed using MassLynx software, version 4.1, with internal standard calibration and quantification to external standard curves. Samples were extracted and analyzed in technical duplicates. When prostaglandin concentrations were below the lower limit of quantification (200 pg/mL), they were given an arbitrary value that equals to 10% of the lowest quantified standard.

### Extraction of RNA from human tendons followed by complementary DNA synthesis and RT-qPCR

RNA isolation and real-time quantitative polymerase chain reaction (RT-qPCR) on tendon samples was performed according to previously described protocol [18]. Gene signatures consisted of a panel of genes using Qiagen validated human primers including *PTGIS* (PGIS, QT00047747), *PTGIR* (IP receptor, Q00072807), *PTGS2* (COX-2, QT00040586), *PTGS1* (COX-1, QT00210280), *PTGES* (mPGES-1, QT00208607), *ACTB (*β-actin*,* QT00095431), and *GAPDH* (GAPDH, QT00079247). The reaction efficiency was calculated by measuring the Ct values for both sets of genes in a cDNA mix dilution series and applying the following formula: Efficiency = 10(− 1/slope) − 1 as previously described [25]. Duplicate reactions for each gene were run on a ViiA7 qPCR machine (Applied Biosystems), and results were calculated using the DDCt method using reference genes for human *ACTB* (β-actin) and *GAPDH* (GAPDH). Results were consistent using these reference genes, and data are shown normalized to β-actin.

### Statistical analysis

Statistical analyses were performed using GraphPad Prism 6 (GraphPad Software). For the prostaglandin data in tendon cells treated with IL-1β and for the mRNA data in tendon biopsies, normality was tested using the Shapiro-Wilk normality test, and the Kruskal-Wallis test with pairwise post-hoc Mann-Whitney *U* test was used to test for significant difference. For the effect of CIII on prostaglandin production in tendon cells treated with IL-1β, a paired *t* test was performed. Statistical significance was set to *p* < 0.05.

## Results

### Diseased tendon tissues highly express prostaglandin biosynthetic enzymes

**Fig. 1:**
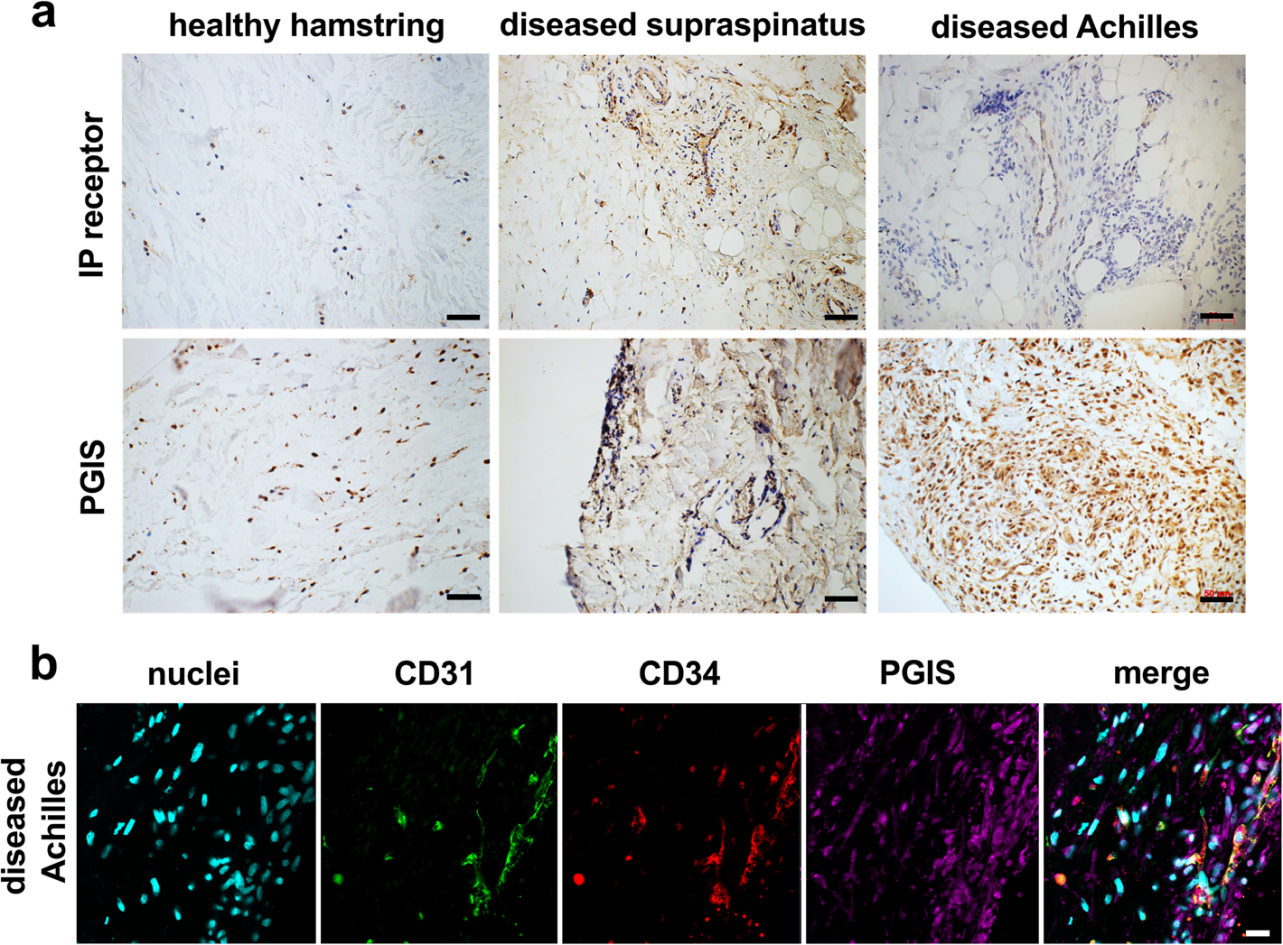
Expression of PGIS in healthy and diseased tendon tissues. **a** Representative immunohistochemistry staining showing expression of IP receptor and PGIS in tendon tissues. Immunostaining (brown) nuclear counterstain is hematoxylin. Scale bar, 50 μm. **b** Representative confocal immunofluorescence images showing staining of cell nuclei (POPO1, cyan), CD31 (green), CD34 (red), and PGIS (purple) in diseased Achilles tendon tissues. Scale bar, 20 μm

**Fig. 2:**
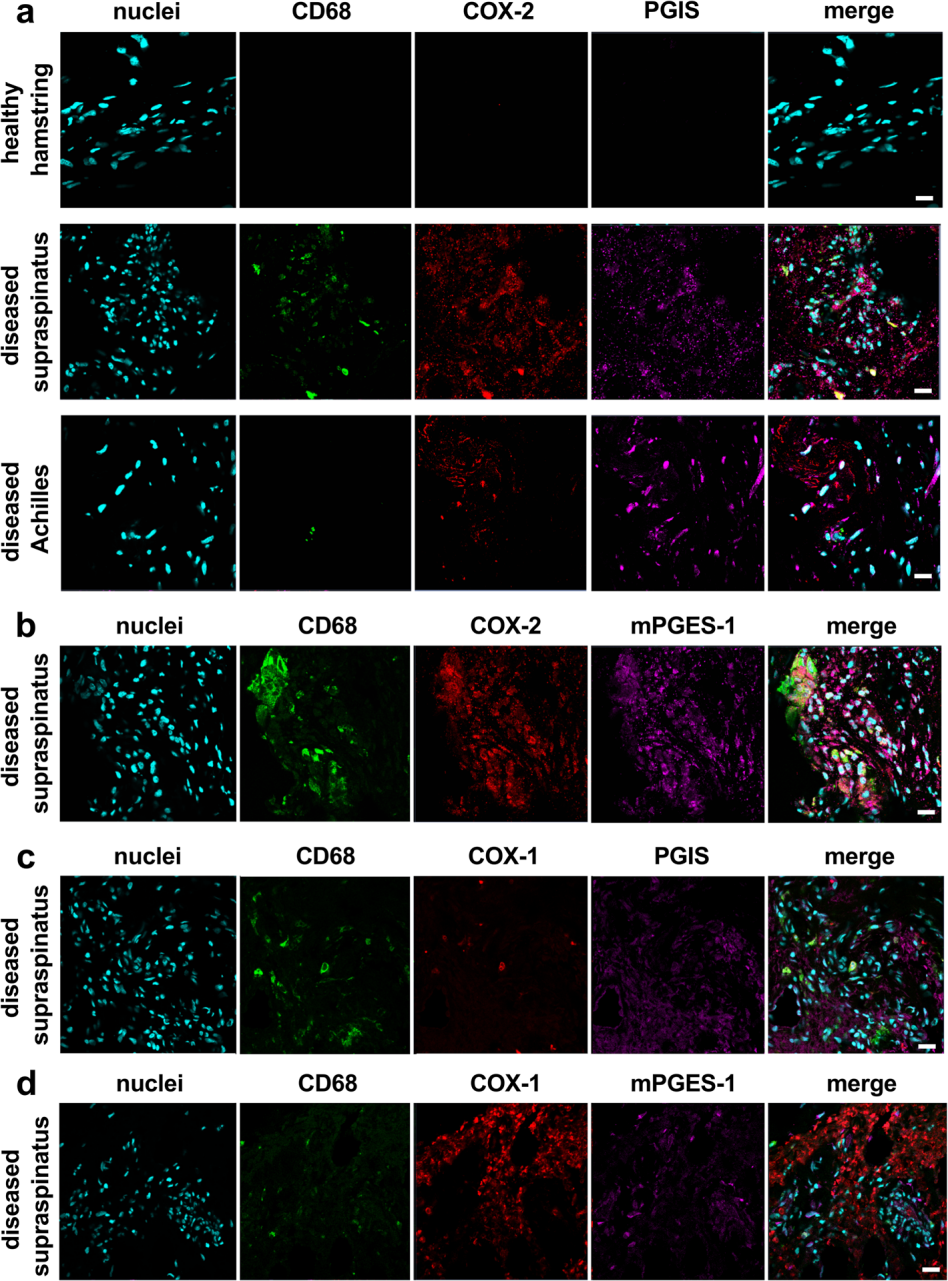
Expression of enzymes implicated in prostaglandin synthesis in diseased tendon tissues. **a**–**d** Representative immunofluorescence images showing staining of cell nuclei (POPO-1, cyan) and macrophages (CD68, green) with **a** expression of COX-2 (red) and PGIS (violet), **b** expression of COX-2 (red) and mPGES-1 (violet), **c** expression of COX-1 (red) and PGIS (violet), and **d** expression of COX-1 (red) and mPGES-1 (violet). Immunostaining in panel **b**–**d** are diseased supraspinatus tendon tissues. Scale bar, 20 μm

Immunostaining of healthy and diseased tendon tissues was performed to identify enzymes involved in the biosynthesis of prostaglandins. Sections of diseased supraspinatus and Achilles tendons showed increased vascularity and cellularity compared to healthy comparator hamstring tendon sections, in line with previous findings [18, 26]. Diseased tendons showed increased IP receptor and PGIS expression compared to healthy tendons (Fig. 1a). Immunostaining was profound in diseased supraspinatus compared to diseased Achilles tendons. In diseased tendons, PGIS expression was identified in vascular regions (CD31^pos^CD34^pos^), although this protein was highly expressed by CD31^neg^CD34^neg^ cells with fibroblast-like morphology, identified to be resident tendon stromal cells (Fig. 1b). PGIS also co-localized with CD68 and COX-2 in diseased tendons (Fig. 2a). Having demonstrated that diseased tendon tissues expressed prostanoid synthetic enzymes COX-2 and PGIS, we next investigated the expression of additional prostanoid synthetic enzymes in diseased and healthy tendon tissues. Diseased tendons highly expressed mPGES-1 that co-localized with COX-2 (Fig. 2b, Additional file 1: Figure S1). mPGES-1 expression was identified on both CD68^pos^ and CD68^neg^ cells. Diseased tendons also expressed COX-1 (Fig. 2c, d).

### PGIS co-localizes with markers of stromal fibroblast activation and nociceptive neuromodulation

**Fig. 3:**
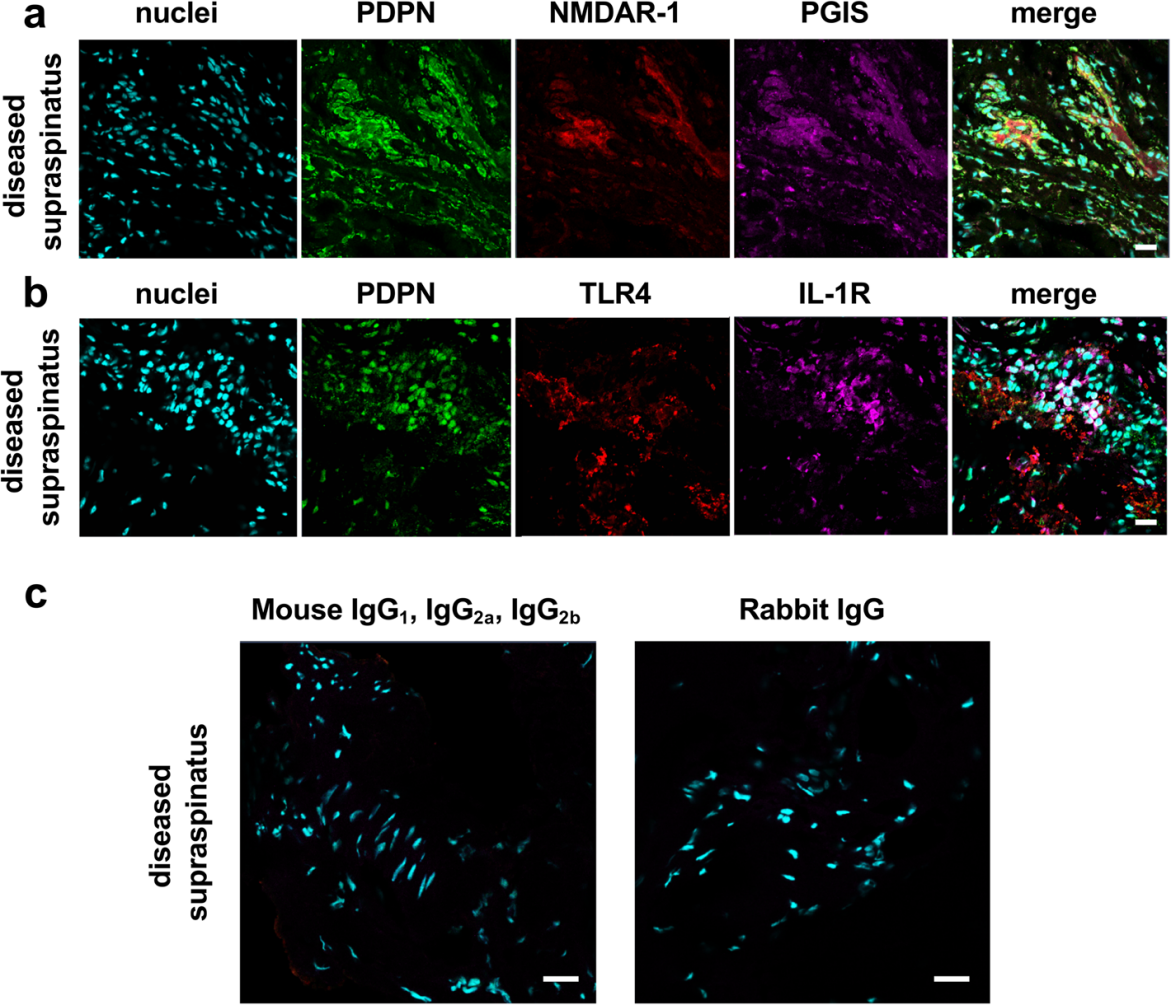
Expression of fibroblast activation marker Podoplanin (PDPN) and nociceptive neuromodulator NMDAR-1 in diseased supraspinatus tendon tissues. **a**–**b** Representative immunofluorescence images showing staining of cell nuclei (POPO-1, cyan) and PDPN (green) with **a** expression of NMDAR-1 (red) and PGIS (violet) and **b** expression of TLR4 (red) and IL-1R (violet). **c** Representative confocal immunofluorescence images showing merged image of diseased tendon sections stained with isotype control antibodies for mouse IgG1, IgG2a, and IgG2b and rabbit IgG fractions. Cyan represents POPO-1 nuclear counterstain. Scale bar, 20 μm

We next investigated if tendon cells expressing PGIS demonstrated activation of biological pathways underpinning inflammation and pain processes. Immunostaining of diseased supraspinatus tendons identified PGIS-immunopositive cells expressed PDPN, a marker of stromal fibroblast activation (Fig. 3a). PGIS-immunopositive cells co-expressed the nociceptive neuromodulator *N*-methyl-d-aspartate receptor subunit 1 (NMDAR-1), implicated in pain perception (Fig. 3a). Tendon cells in sections from diseased supraspinatus tissues showed a pro-inflammatory phenotype also expressing Toll-like receptor 4 (TLR4) and IL-1 receptor (IL-1R) (Fig. 3b). Collectively, these findings suggest that resident stromal fibroblasts highly express markers of inflammation and pain pathways during tendon disease. Under inflammatory conditions, tendon cells also express enzymes implicated in prostacyclin biosynthesis. Isotype control staining was performed on diseased tendon tissues (Fig. 3c).

### Tendon-derived stromal cells isolated from diseased supraspinatus show enhanced prostaglandin biosynthesis

**Fig. 4:**
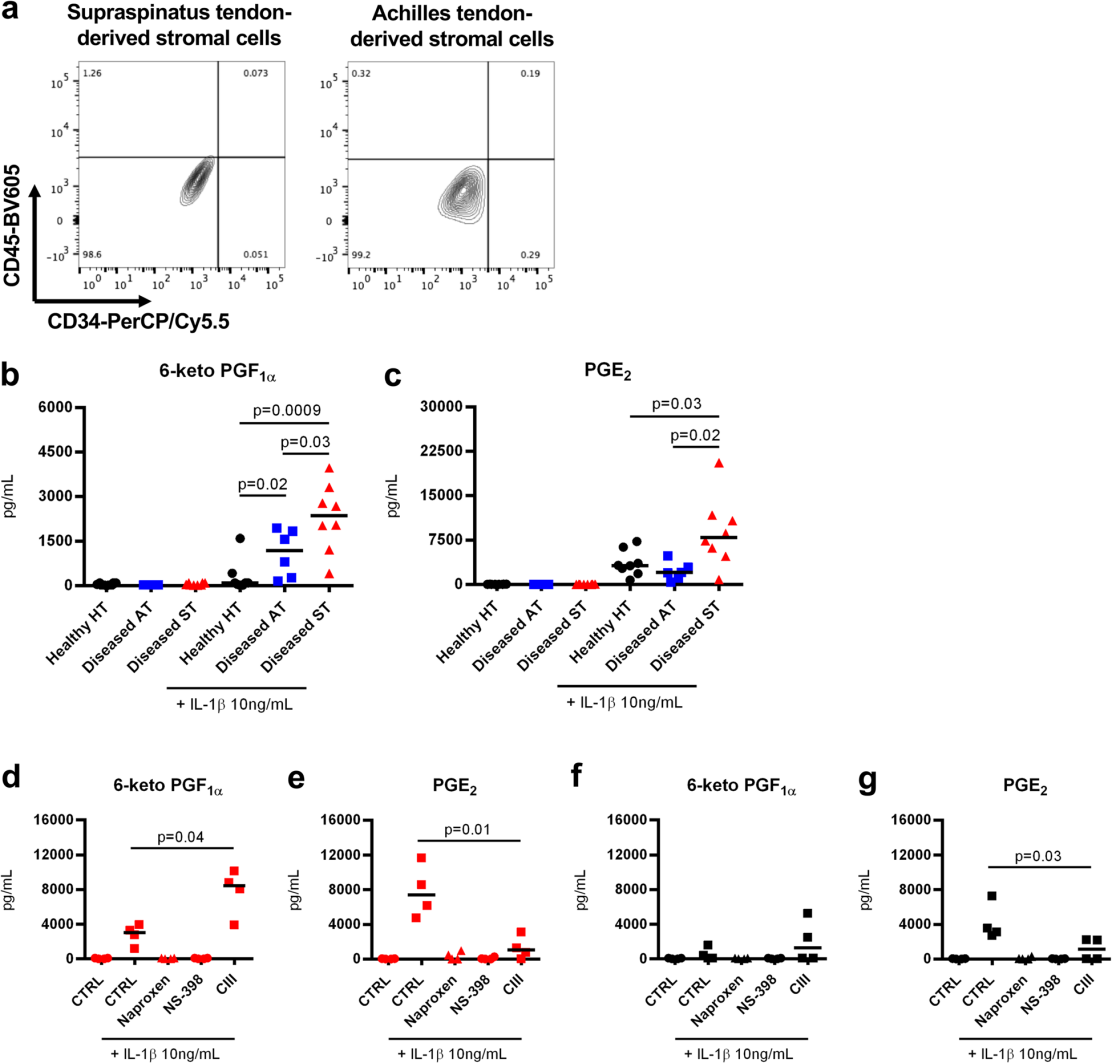
Prostaglandin production in tendon-derived stromal cells and expression of prostaglandin synthetic enzymes in tendon biopsies. **a** Representative FACS contour plots showing that cultured tendon-derived stromal cells do not express markers of endothelial cells (CD45) or leukocytes (CD34). **b**, **c** Tendon-derived cells from healthy hamstring tendons (HT; *n* = 8), diseased Achilles tendons (AT; *n* = 6), and diseased supraspinatus tendons (ST; *n* = 8) were cultured and treated with medium only or medium containing 10 ng/mL IL-1β for 24 h. Prostaglandins in supernatants were extracted and quantified using LC-MS/MS. Lower limit of quantification was 200 pg/mL. Prostacyclin production was quantified by measuring stable metabolite 6-keto PGF_1α_. The bars show median values. Statistical significance was calculated using Kruskal-Wallis test with pairwise post-hoc Mann-Whitney *U* test. **d**–**g** Effect on prostaglandin profile upon inhibition of COX or mPGES-1 in IL-1β-treated tendon cells. Diseased (*n* = 4, **d**, **e**) and healthy (*n* = 4, **f**, **g**) tendon-derived cells were treated with vehicle, IL-1β alone (10 ng/mL), or IL-1β together with either dual COX-1/2 inhibitor naproxen (10 μM), selective COX-2 inhibitor NS-398 (10 μM), or selective mPGES-1 inhibitor CIII (10 μM) for 24 h. Bars show median values. Statistical significance was calculated using paired *t* test

Using cultures of tendon-derived stromal cells from healthy donors and patients with tendinopathy, we next investigated differences in prostaglandin profiles between healthy and diseased cells before and after treatment with IL-1β (10 ng/mL). Cells isolated from tendons at passage 1 or 2 did not express markers for leukocytes (CD45) or endothelial (CD34) cells (Fig. 4a) [19]. Only diseased tendon cells were sensitive to IL-1β for production of prostacyclin, as reflected by stable metabolite 6-keto PGF_1α_ (Fig. 4b). Prostacyclin production (mean ± SEM) was higher in tendon stromal cells isolated from diseased supraspinatus (2300 ± 400 pg/mL, *n* = 8) compared to those in diseased Achilles (1100 ± 300 pg/mL, *n* = 6, *p* = 0.03). Moreover, both healthy and diseased tendon cells produced PGE_2_ upon treatment with IL-1β for 24 h (Fig. 4c). PGE_2_ production was higher in cells isolated from diseased supraspinatus (8800 ± 2100 pg/mL, *n* = 8) compared to diseased Achilles (2200 ± 600 pg/mL, *n* = 6, *p* = 0.02) and healthy hamstring (3600 ± 800 pg/mL, *n* = 8, *p* = 0.03).

### mPGES-1 inhibition potentiates prostacyclin production in diseased tendon-derived stromal cells

Tendon-derived cells from healthy and diseased tendons were treated with inhibitors of COX or mPGES-1 to investigate their effects on prostaglandin production. The non-selective COX inhibitor naproxen (10 μM) and the selective COX-2 inhibitor NS-398 (10 μM) both blocked IL-1β-induced prostacyclin production by > 96% in diseased tendon cells (Fig. 4d). Naproxen and NS-398 also inhibited IL-1β-induced PGE_2_ production by > 96% in both diseased (Fig. 4e) and healthy tendon stromal cells (Fig. 4g). The selective mPGES-1 inhibitor Compound III (CIII, 10 μM) reduced IL-1β-induced PGE_2_ production in diseased and healthy tendon stromal cells by 83% (*p* = 0.01, Fig. 4e) and 70% (*p* = 0.03, Fig. 4g), respectively. Reduced PGE_2_ levels coincided with an increased prostacyclin production by 240% in diseased (*p* = 0.04, Fig. 4d) but not healthy tendon cells (Fig. 4f).

### Prostacyclin synthase is implicated in the resolution of tendon pain

**Fig. 5:**
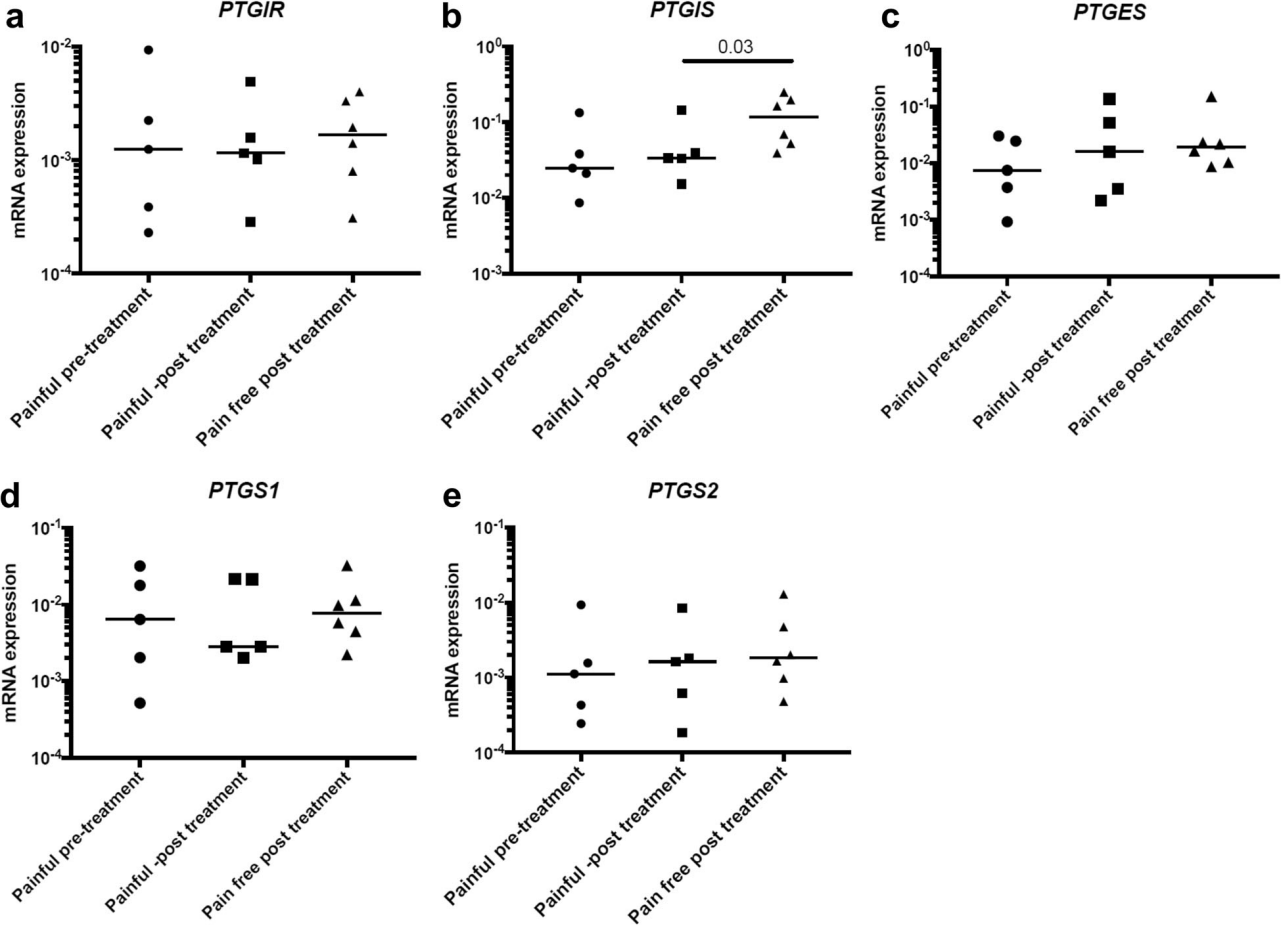
Expression of selected genes in biopsies from patients with supraspinatus tendinopathy before and after arthroscopic subcomial decompression. The selected genes were *PTGIR* (IP receptor, **a**), *PTGIS* (PGIS, **b**), *PTGES* (mPGES-1, **c**), *PTGS1* (COX-1, **d**), and *PTGS2* (COX-2, **e**). Gene expression is normalized to β-actin, and the bars show median values. Statistical significance was calculated using Kruskal-Wallis test with pairwise post-hoc Mann-Whitney *U* test

To investigate if prostacyclin or PGE_2_ are associated with tendon pain perception, we next investigated mRNA expression of IP receptor (*PTGIR*) and prostanoid synthetic enzymes PGIS (*PTGIS*), mPGES-1 (*PTGES*), COX-1 (*PTGS1*), and COX-2 (*PTGS2*) in tissue biopsies from patients with resolved (*n* = 6) or persistent (*n* = 5) supraspinatus tendinopathy. Biopsies from resolved (pain-free) patients showed increased *PTGIS* mRNA expression compared to patients with persistent tendon disease (*p* = 0.03). *PTGIR*, *PTGES*, *PTGS1*, or *PTGS2* mRNA expression did not differ between pain-free post-treatment and painful post-treatment patients (Fig. 5).

## Discussion

We provide new insight into the mechanisms underlying inflammation and pain in disorders affecting musculoskeletal soft tissues such as tendons. We found that diseased tendon tissues show increased expression of key enzymes implicated in prostaglandin biosynthesis, including PGIS, COX-2, and mPGES-1. The difference in PGIS expression is reflected in an in vitro model of tendon inflammation, as only diseased and not healthy tendon-derived cells produced prostacyclin in response to IL-1β treatment. These cells are non-endothelial, non-immune cells (CD31^neg^CD34^neg^CD45^neg^), suggesting diseased tendon fibroblasts show prostacyclin synthetic capacity. Both healthy and diseased tendon stromal cells produced PGE_2_ upon IL-1β treatment. To investigate if prostacyclin production correlated with pain in tendon disease, we investigated expression of PGIS (*PTGIS*) in tendon biopsies from patients that remained symptomatic compared to those whose symptoms resolved. Interestingly, we found that *PTGIS* expression was increased in tendon biopsies from pain-free (resolved) patients, suggesting that increased capacity for prostacyclin production is not associated with tendon pain. Collectively, our results identify resident tendon stromal cells as an important cell type implicated in sustaining inflammation. These findings also highlight the divergent roles of prostaglandins in tendon disease, identifying a potentially protective role for prostacyclin.

Prostacyclin is well characterized as a regulator of vascular homeostasis and thrombosis prevention but it is also a mediator of edema and pain [27]. Prostacyclin is the main prostaglandin found in synovial fluid of patients with rheumatoid arthritis [28], and it has been demonstrated in two arthritis models that IP-deficient mice have reduced disease severity [29, 30]. Early work by Murata et al. showed that mice lacking the IP receptor have reduced paw edema and pain sensation when challenged with carrageenan but the mice displayed increased propensities towards thrombosis [13]. Sugita et al. have reported that simultaneous targeting of prostacyclin and PGE_2_ signaling or production was needed for analgesic effect in pain models of mice, since celecoxib exhibited this effect while mPGES-1 inhibition or IP receptor antagonist alone did not [31]. The vasodilating role of prostacyclin is evident in pulmonary atrial hypertension and Raynaud’s syndrome, where infusion of prostacyclin analogues are used as treatments [32, 33]. The cardiovascular hazard seen with NSAIDs that targets COX-2 is at least partly driven by the reduction in anti-thrombotic prostacyclin [15]. Collectively, prostacyclin can therefore be attributed detrimental (pro-inflammatory and pain-mediating) and beneficial (vasodilating and anti-thrombotic) actions depending on the clinical and tissue-specific context. Our experiments showed that PGIS expression and prostacyclin production were increased in diseased tendon tissues and cells and that increased expression of PGIS (*PTGIS*) in tendon biopsies from patients after surgical treatment correlated with resolved symptoms of tendon disease. Our data suggest that prostacyclin does not correlate with pain in tendon disease. Instead, we propose that the prostacyclin production may be a protective response to promote vascularization and minimize thrombosis at the site of injury.

**Fig. 6:**
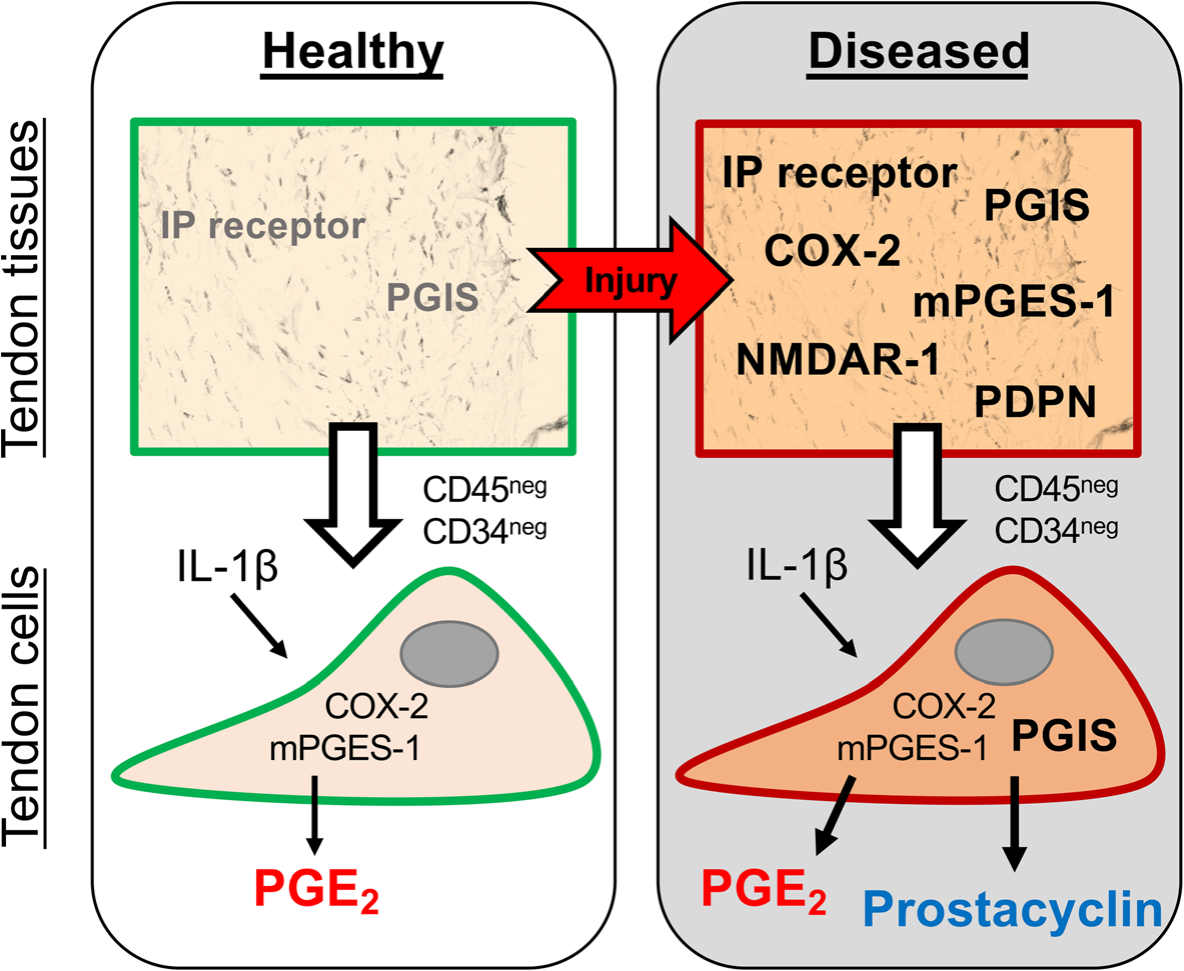
Schematic summarizing the key findings. Tendon injury induces a pro-inflammatory state whereby diseased tendon tissues highly express IP receptor, PGIS, COX-2, mPGES-1, NMDAR-1, and PDPN. Tendon stromal cells (CD45^neg^CD34^neg^) from diseased tendon tissues produce prostacyclin and PGE_2_ upon treatment with IL-1β, while healthy tendon cells only produce PGE_2_

It was recently reported that tendon stromal cells isolated from healthy volunteers and patients with tendon disease can produce numerous lipid mediators [34]. Here, we report that diseased supraspinatus tendon cells produce more PGE_2_ than healthy hamstring tendon cells after IL-1β treatment. Conversely, diseased Achilles tendon cells did not produce significantly elevated levels of PGE_2_. Our results show that diseased supraspinatus tendon cells may be primed after exposure to an inflammatory environment due to injury and then hyper-respond on exposure to subsequent inflammatory stimuli (Fig. 6). This finding may be attributable to the temporal effects of the stage of disease between supraspinatus and Achilles tendon disorders. Furthermore, fibroblasts from different joints maintain their phenotype, positional memory, and topographic differentiation ex vivo [35]. This variation in IL-1β-induced PGE_2_ synthetic capacity between functionally distinct tendons may be attributable to epigenetically driven anatomical diversity between supraspinatus and Achilles tendons, which is also a feature of synovial fibroblasts in inflammatory arthritis [36].

The functional role of PGE_2_ in tendon biology is not fully understood, since studies have shown both beneficial and detrimental associations. Khan et al. have demonstrated that repeated injection of PGE_2_ into rabbit patellar tendons caused degenerative processes (collagen fibril disorganization and thinner collagen fibril diameter) [37], while Ferry et al. have reported that similar injections improved mechanical properties of tendons [38]. One in vitro study showed that low PGE_2_ concentrations (< 1 ng/mL) promote cell proliferation while high PGE_2_ concentrations (> 1 ng/mL) induced differentiation of human tendon stem cells into non-tenocytes [39]. Moreover, PGE_2_ generated during inflammation is a requirement to trigger endogenous tissue resolution responses [40]. The biological consequence of PGE_2_ in the tendon environment may therefore be dependent on the duration of production and the endogenous concentration. Nonetheless, PGE_2_ is a key mediator of inflammation and an important mediator of pain, edema, and cartilage erosion typically observed in rheumatoid arthritis patients [41, 42]. In line with this, several studies support that genetic deletion or pharmacological inhibition of mPGES-1 is protective in inflammatory disease models [24, 43–45]. Our results showed that mPGES-1 is highly expressed in diseased tendon tissues and that this does not correlate with tendon pain. We conclude that inflammation in supraspinatus tendon disease is at least partly driven by the COX-2/mPGES-1/PGE_2_ pathway.

NSAIDs are widely used for their anti-inflammatory and analgesic effect but they are also associated with adverse effects in tendon disease including tendon rupture [4–6] and impaired healing [7, 8]. Given that COX-2 inhibitors dampen protective responses regulating resolution of inflammation [46, 47], targeting mPGES-1 is a potential therapeutic strategy to regulate inflammation without impeding resolution of tendon inflammation. We investigated how COX and selective mPGES-1 inhibition affected the prostaglandin profiles of IL-1β-treated healthy and diseased tendon cells. The non-selective COX inhibitor naproxen and the selective COX-2 inhibitor NS-398 blocked both PGE_2_ and prostacyclin production. This suggests that COX-2 is the dominant source for PGH_2_ generation in tendon cells during inflammatory condition. Moreover, treatment with selective mPGES-1 inhibitor CIII reduced PGE_2_ production and instead increased prostacyclin formation. This can be explained by shunting of PGH_2_ to PGIS when mPGES-1 is inhibited, an effect that has been observed in studies on genetic deletion [48, 49] or pharmacological inhibition [50, 51] of mPGES-1. Multiple studies support that inhibition of mPGES-1 activity by genetic deletion or pharmacological inhibition is protective in inflammation [24, 43–45] and that shunting towards prostacyclin is regarded as a cardioprotective effect [45, 52, 53]. Given that NSAIDs block the production of both prostacyclin and PGE_2_ in tendon-derived cells and that the use of NSAIDS are associated with reduced healing in tendon disease, this supports the concept of prostacyclin as a protective factor. The use of selective mPGES-1 inhibitor would then be a superior treatment based on the reduction in pro-inflammatory PGE_2_ and promotion of protective prostacyclin.

We acknowledge that there are potential study limitations in using hamstring tendon as comparator to diseased supraspinatus and Achilles tendons, including differences in donor age, sex, and tendon type. However, hamstring tendons collected from healthy donors without history of tendinopathy is preferred over cadaveric supraspinatus and Achilles tendon, which can be affected by post-mortem changes and where little is known about the health status of the tissue.

## Conclusions

To our knowledge, the prostacyclin axis has not been investigated in the context of tendon disease. We found diseased tendon stromal cells highly expressed COX-2, PGIS, mPGES-1, and NMDAR1, synonymous with a pro-inflammatory and nociceptive phenotype. Given that these cells constitute the majority cell type in tendons, future therapies should address the pathobiology of these cells. Our results suggest that PGE_2_ sustains inflammation and pain while prostacyclin may have a protective role in human tendon disease. Further investigation of the prostacyclin axis and the COX-2/mPGES-1/PGE_2_ pathway may inform future therapeutic strategies to treat musculoskeletal soft tissue disorders, based on selective manipulation of prostaglandin production.

## Additional file

Additional file 1: **Figure S1.** mPGES-1 immunostaining in diseased Achilles tendon tissue. Representative image showing immunostaining (brown), nuclear counterstain is hematoxylin. Scale bar, 50 μm. (TIF 6981 kb)

## Abbreviations

ASAD: Arthroscopic subacromial decompression
BSA: Bovine serum albumin
CIII: Compound III
COX-1/2: Cyclooxygenase-1/2
DAB: 3,3′-Diaminobenzidine
FA: Formic acid
GAPDH: Glyceraldehyde-3-phosphate dehydrogenase
IL-1R: Interleukin-1 receptor
IL-1β: Interleukin-1β
IP: Prostacyclin receptor
LC-MS/MS: Liquid chromatography tandem mass spectrometry
mPGES-1: Microsomal prostaglandin E synthase-1
NF-κB: Nuclear factor kappa beta
NMDAR-1: *N*-Methyl-d-aspartate receptor subunit 1
NSAIDs: Non-steroidal anti-inflammatory drugs
OSS: Oxford Shoulder Score
PDPN: Podoplanin
PGE_2_: Prostaglandin E2
PGIS: Prostacyclin synthase
RT-qPCR: Real-time quantitative polymerase chain reaction
SPE: Solid-phase extraction
TLR4: Toll-like receptor 4
VCAM-1: Vascular cell adhesion molecule-1
