# Supplementary figures and images for: Divergent roles of prostacyclin and PGE2 in human tendinopathy
Source: Arthritis Res Ther. 2019 Mar 13;21:74. doi: 10.1186/s13075-019-1855-5 (PMC6416900; doi:10.1186/s13075-019-1855-5)

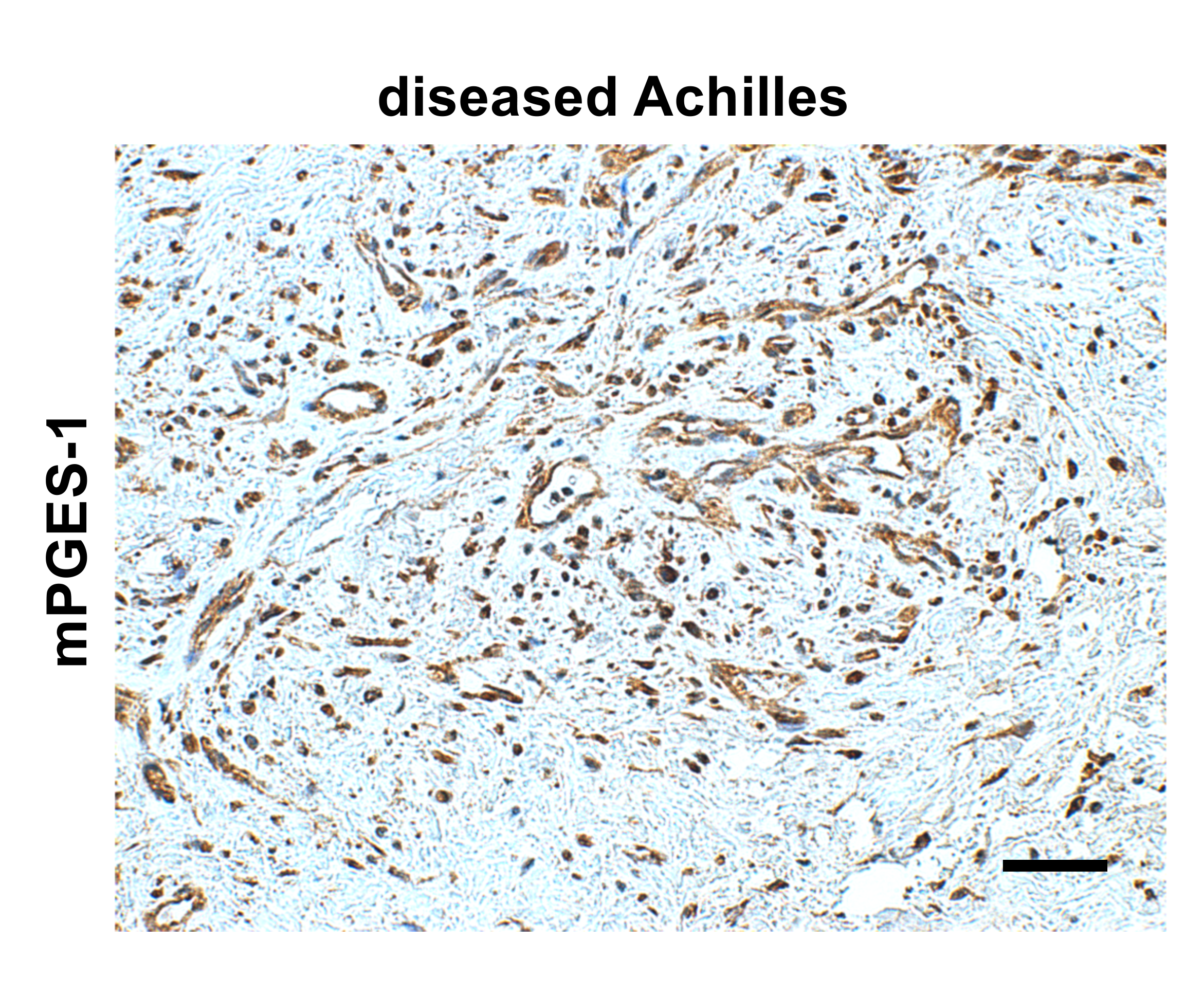

Supplement: Supplementary file 1 — Figure S1. mPGES-1 immunostaining in diseased Achilles tendon tissue. Representative image showing immunostaining (brown), nuclear counterstain is hematoxylin. Scale bar, 50 μm. (TIF 6981 kb) [file 13075_2019_1855_MOESM1_ESM.tif]
